# How to pause fertility

**DOI:** 10.7554/eLife.97432

**Published:** 2024-04-09

**Authors:** Aleisha M Moore

**Affiliations:** 1 Department of Biological Sciences, Brain Health Research Institute, Kent State University Kent United States

**Keywords:** lactational amenorrhea, kisspeptin neurons, GnRH pulse generator, fertility, Mouse

## Abstract

Prolactin suppresses the ovarian cycles of lactating mice by directly repressing the activity of a cell population known as kisspeptin neurons.

**Related research article** Hackwell ECR, Ladyman SR, Clarkson J, McQuillan HJ, Boehm U, Herbison AE, Brown RSE, Grattan DR. 2024. Prolactin mediates a lactation-induced suppression of arcuate kisspeptin neuronal activity necessary for lactational infertility in mice. *eLife* **13**:RP94570. doi: 10.7554/eLife.94570.

Milk production requires a lot of energy from the body – and so does taking care of newly born cubs, kits, pups or babies. Many mammals have evolved to suppress fertility during lactation, preventing the physical burdens of pregnancy from occurring at such a demanding time (Smith, 1978a). This translates into a cessation of menstrual or oestrous cycles, which are under the influence of a brain region known as the hypothalamus.

During a fertile cycle, complex cell networks converge to control a small population of neurons in the hypothalamus that produce gonadotropin-releasing hormone (or GnRH) in a pulsatile manner. This signal stimulates the pituitary gland beneath the brain to release pulses of luteinizing hormone (or LH), which travels through the bloodstream to control ovary function. During lactation the secretion of both GnRH and LH is suppressed, resulting in absent ovarian cycles (Smith, 1978a). However, the precise underlying mechanism controlling this type of infertility – known as lactational amenorrhea – is still not fully understood.

After birth, suckling is a stimulus that induces large quantities of the hormone prolactin to be released from the pituitary gland (Hwang et al., 1971). Prolactin acts on a variety of central and peripheral tissues to stimulate milk production, regulate energy levels, and, as animal studies show, reduce stress and promote maternal behavior (Georgescu et al., 2021). Elevated prolactin levels are also thought to suppress LH during lactation, as they are a well-established cause of both male and female infertility; in fact, treating rodents with prolactin is enough to suppress LH secretion (Brown et al., 2019). However, other mechanisms could also be at play, such as neural pathways that are directly activated by the suckling or the levels of other hormones being increased (Smith, 1978b). Delineating the relative importance of prolactin in suppressing LH and fertility during lactation has therefore been a challenge so far. Now, in eLife, David Grattan of the University of Otago and colleagues – including Eleni Hackwell as first author – report new insights into how prolactin acts on the neural networks controlling ovarian cycles (Hackwell et al., 2024).

The researchers, who are based at institutes in New Zealand, Germany and the United Kingdom, started by generating mice in which the prolactin receptor had been genetically deleted from neurons. LH pulses and estrous cycles were prematurely re-established during early lactation in these animals, supporting the hypothesis that prolactin receptors in the brain are required to suppress GnRH/LH release and fertility during lactation. However, only a minute proportion of GnRH neurons express the prolactin receptor, suggesting that another neural population is involved ‘upstream’ to mediate prolactin-induced infertility (Kokay et al., 2011).

To explore this possibility, Hackwell et al. focused on a group of neurons in the arcuate nucleus of the hypothalamus which have recently been defined as the long sought-after ‘GnRH pulse generator’. These cells produce an essential regulator of the reproductive axis known as kisspeptin, and they exhibit synchronized activity immediately prior to an LH pulse (Clarke et al., 2015; Clarkson et al., 2017). Inhibiting these neurons or deleting kisspeptin is sufficient to suppress GnRH/LH pulses; conversely, stimulating kisspeptin release from these cells can increase GnRH neuron activity and induce an LH pulse (Clarkson et al., 2017; Nagae et al., 2021).

The team used fiber photometry to record the activity of arcuate kisspeptin neurons in freely behaving mice that had been genetically altered to carry a marker which glows strongly when these cells are active (Figure 1A). This helped Hackwell et al. establish that these neurons show episodes of activity synchronous with LH pulses in virgin animals, and are silenced during pregnancy and lactation (Figure 1B). Strikingly, episodic LH activity and ovarian cycles returned during early lactation in mice in which the prolactin receptor had been conditionally deleted from kisspeptin cells (Figure 1B). Fertility was re-established even earlier when the prolactin receptor was absent in all neurons. Since this knockout approach did not affect other mechanisms hypothesized to lower LH during lactation, such as the neurogenic effect of suckling, these findings strongly support prolactin as the primary factor that induces lactational infertility in mice.

**Figure 1.**
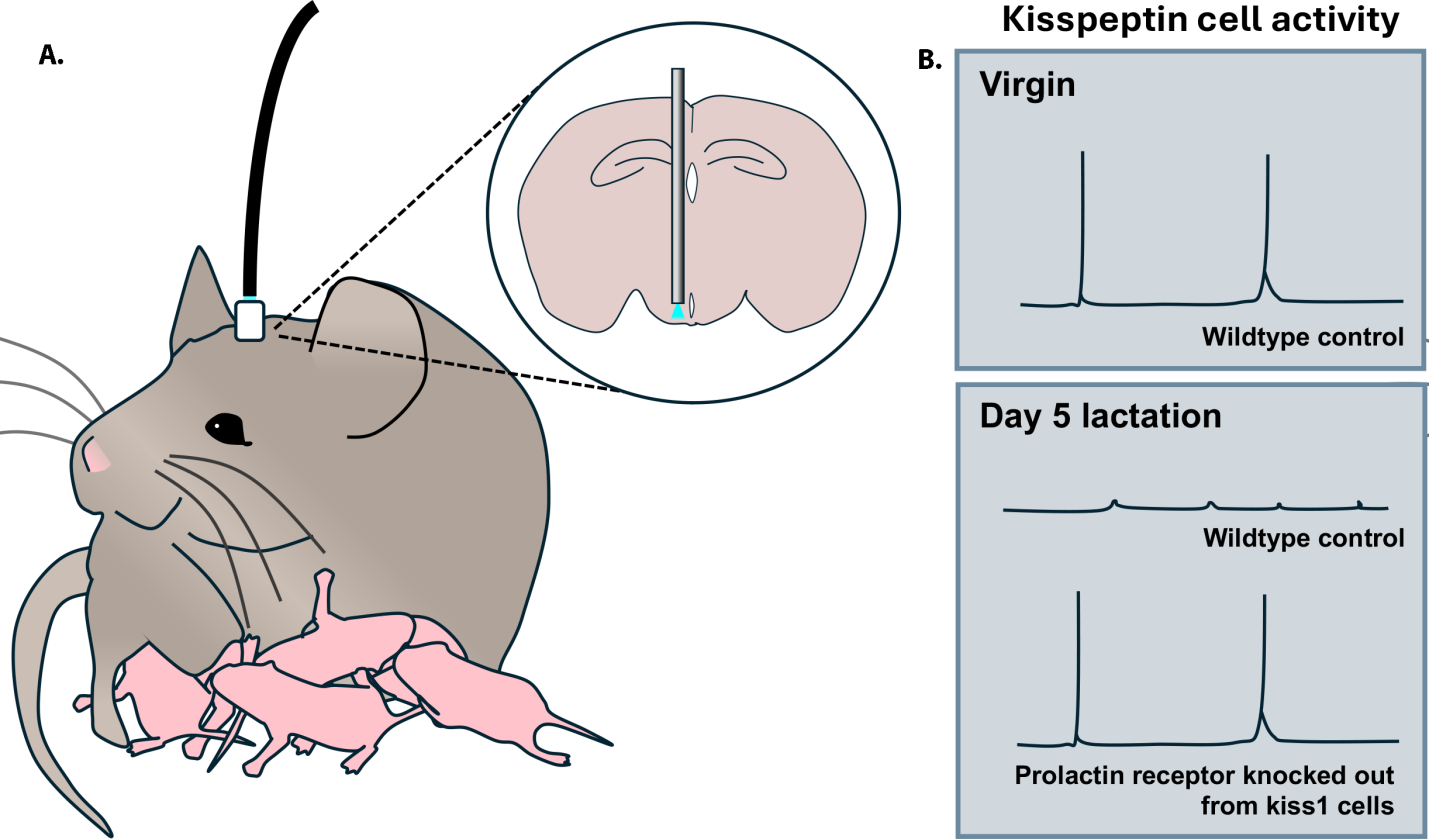
Activity of arcuate kisspeptin neurons over different reproductive states. (**A**) Mice were genetically manipulated so that kisspeptin neurons in their arcuate nucleus produced a fluorescent calcium indicator that emits light (blue) when the cell is active. This signal can be detected via an optic fiber implanted in the animal (grey rod in inset) and relayed to a photometry system (black cable). (**B**) Episodic activity of kisspeptin neurons can be detected in virgin mice, but not during lactation. In contrast, such activity is present in lactating mice in which the prolactin receptor has been genetically deleted from kisspeptin (kiss1) cells.

Overall, the work by Hackwell et al. highlights arcuate kisspeptin neurons as a primary site of prolactin action during lactation, significantly advancing our understanding of the mechanisms underlying lactational amenorrhea. By revealing that ovarian cycles return earlier when prolactin receptors are absent from all neurons, it also suggests that other prolactin-sensitive cell populations are involved; promising candidates include a rostral kisspeptin population responsible for generating the LH surge that drives ovulation, but further investigation is required.
